# Decreased calpain activity in chronic myeloid leukemia impairs apoptosis by increasing survivin in myeloid progenitors and xiap1 in differentiating granulocytes

**DOI:** 10.18632/oncotarget.16884

**Published:** 2017-04-06

**Authors:** Weiqi Huang, Ling Bei, Elizabeth E. Hjort, Elizabeth A. Eklund

**Affiliations:** ^1^ The Feinberg School at Northwestern University, Chicago, IL, USA; ^2^ Jesse Brown VA Medical Center, Chicago, IL, USA

**Keywords:** leukemia, Calpain, apoptosis, myeloid, survivin

## Abstract

Chronic Myeloid Leukemia (CML) is characterized by translocations between chromosomes 9 and 22, resulting in expression of Bcr-abl oncogenes. Although the clinical course of CML was revolutionized by development of Bcr-abl-directed tyrosine kinase inhibitors (TKIs), CML is not cured by these agents. Specifically, the majority of subjects relapsed in clinical trials attempting TKI discontinuation, suggesting persistence of leukemia stem cells (LSCs) even in molecular remission. Identifying mechanisms of CML-LSC persistence may suggest rationale therapeutic targets to augment TKI efficacy and lead to cure. Apoptosis resistance is one proposed mechanism. In prior studies, we identified increased expression of Growth Arrest Specific 2 (Gas2; a Calpain inhibitor) in Bcr-abl^+^ bone marrow progenitor cells. A number of previously described Calpain substrates might influence apoptosis in CML, including βcatenin and the X-linked Inhibitor of Apoptosis Protein 1 (Xiap1). We previously found Gas2/Calpain dependent stabilization of βcatenin in CML, and increased expression of βcatenin target genes, including Survivin (also an IAP). In the current work, we investigate contributions of Survivin and Xiap1 to Fas-resistance in Bcr-abl^+^ bone marrow cells. Inhibitors of these proteins are currently in clinical trials for other malignancies, but a role for either IAP in CML-LSC persistence is unknown.

## INTRODUCTION

CML is characterized by translocations between chromosomes 9 and 22, resulting in expression of Bcr-abl tyrosine kinase oncogenes [1]. Development of Bcr-abl-directed TKIs increased survival in CML by delaying progression to fatal blast crisis (BC) [2–5]. Unfortunately, additional clinical studies indicated CML is not cured by these agents [6–8]. Specifically, in studies attempting discontinuation of TKI treatment, the majority of CML subjects relapsed, even though they had been in sustained major molecular response. Obtaining a second remission required a longer duration of TKI treatment than at presentation, suggesting LSCs not only persisted, but also expanded, during treatment [6]. Identifying molecular mechanisms responsible for LSC persistence during TKI treatment might indicate potential therapeutic targets to specifically address this population. CML-LSCs are hypothesized to possess intrinsic insensitivity to TKIs, not requiring *BCRABL* gene duplications or point mutations found with overt TKI resistance. One mechanism for this may be relative CML-LSC quiescence in comparison to actively proliferating differentiating CML progenitor cells. Another potential mechanism for CML-LSC persistence during TKI treatment is intrinsic apoptosis-resistance [9–11].

In prior studies, we identified increased expression of Fap1 (Fas-associated phosphatase 1) as a mechanism for Fas-resistance in CML [12–14]. Fap1 interacts with and dephosphorylates Fas, antagonizing Fas-induced apoptosis [15, 16]. We found that inhibiting Fap1, with a blocking peptide or small molecule, delayed development of TKI resistance and prevented progression to blast crisis in a murine CML model [14]. Unfortunately, there are no currently available Fap1 inhibitors appropriate for human clinical trials.

Decreased Calpain activity may also contribute to apoptosis resistance in CML. In prior studies, we found increased expression of the endogenous Calpain inhibitor, Growth Arrest Specific 2 (Gas2) in Bcr-abl^+^ myeloid progenitor cells [17]. Increased Gas2 expression decreased Calpain activity, stabilizing βcatenin protein and increasing its activity in these cells [17]. This had implications for apoptosis resistance, since *BIRC5* (encoding Survivin) is a βcatenin target gene [17]. Survivin is an Inhibitor of Apoptosis Protein (IAP), but does not have a known role in CML-LSC persistence [18]. Xiap1 (another IAP) is also a Calpain substrate not previously implicated in CML-LSC biology [19]. These IAPs are of particular interest because inhibitors for each of them are currently in human clinical trials for solid tumors, but have not been tested in CML [20–23].

We identified involvement of Fap1 and Calpain in apoptosis resistance in CML while investigating mechanisms of leukemia suppression by the Interferon Consensus Sequence Binding Protein (Icsbp, also referred to as Interferon Regulatory Factor 8; Irf8). Gene expression profiling studies identified decreased Icsbp expression in the bone marrow of CML subjects in comparison to normal [24, 25]. Icsbp expression increases with TKI- or interferon-induced remission, falls with development of drug resistance, and is lowest in BC [24, 25]. In murine transplantation experiments, myeloproliferation was decreased and BC delayed in recipients of bone marrow transduced with retroviral vectors to express Bcr-abl + Icsbp in comparison to recipients of bone marrow with Bcr-abl alone [26]. And, Icsbp^−/−^ mice exhibited myeloproliferation with granulocytosis, progressing to BC over time, phenocopying CML [27, 28]. We identified repression of genes encoding Fap1 (*PTPN13*) and Gas2 (*GAS2*) by Icsbp in myeloid progenitor cells [12, 17]. Icsbp also repressed *PTPN13*, but not *GAS2*, in differentiating granulocytes [12, 17]. We found Calpastatin, not Gas2, was the major endogenous Calpain inhibitor in differentiating myeloid cells [17].

Therefore, Calpain activity is regulated by different mechanisms during the course of myelopoiesis. It is also possible Calpain influences different substrates at various points in the process, although this has not been previously investigated. Since CML progenitors re-capitulate many steps of normal granulopoiesis, some Calpain substrates might be relevant to apoptosis resistance of CML-LSCs and others may be more relevant to survival of differentiating progenitors or circulating, mature CML granulocytes. The goal of these studies is to establish the relative contributions of Gas2 and Calpastatin to Calpain inhibition and apoptosis resistance in CML. We will also determine if either Survivin or Xiap1 influence apoptosis in CML-LSCs.

## RESULTS

### Bcr-abl and Icsbp influence expression of Calpastatin in differentiating granulocytes

We first investigated mechanisms that regulate Calpain activity during normal myelopoiesis and in Bcr-abl^+^ cells. We approached this by examining expression of the major endogenous Calpain inhibitors in myeloid cells; Gas2 and Calpastatin [29, 30]. For these experiments, we studied murine bone marrow cells transduced with a retroviral vector to express Bcr-abl or with control vector. To determine if decreased Icsbp expression recapitulated any Bcr-abl effects, we also studied bone marrow cells from mice with constitutive disruption of the *IRF8* gene (Icsbp^−/−^ mice). Since Bcr-abl^+^ Lin^−^Sca1^−^ckit^+/−^CD34^+^CD38^−^ bone marrow cells function as LSCs in murine chronic phase CML models, we studied these cells with or without *ex vivo* granulocyte differentiation with G-CSF [15, 16, 31].

**Figure 1:**
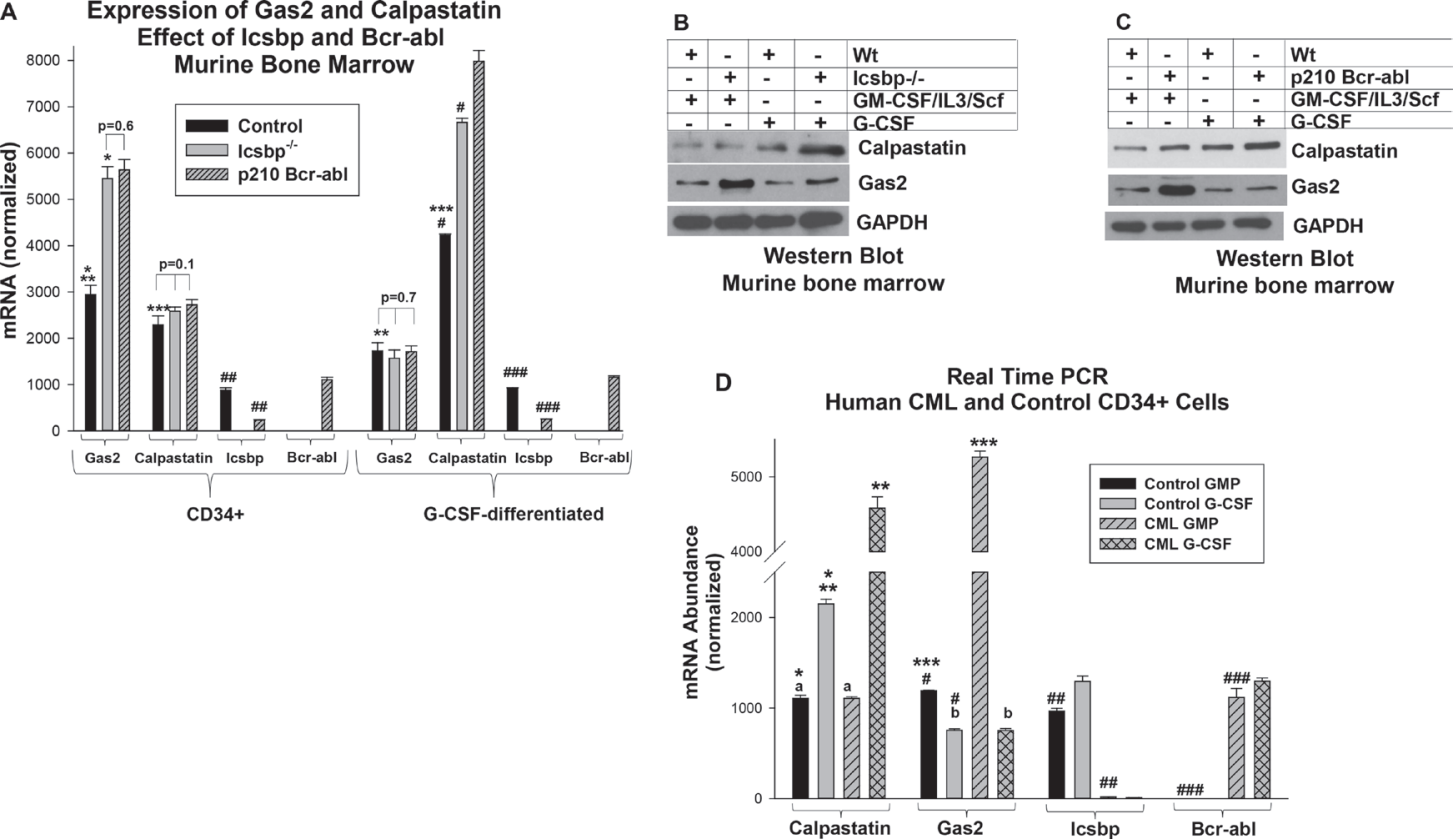
Expression of Gas2 and Calpastatin is increased in CML (**A**) Either Bcr-abl expression or Icsbp knockout increased Gas2 mRNA in myeloid progenitor cells and Calpastatin mRNA in differentiating granulocytes. Bone marrow cells from wild type and Icsbp^−/−^ mice were compared; some wild type cells were transduced with a Bcr-abl-expression vector. Lin^−^CD34^+^ cells were analyzed for Gas2 or Calpastatin mRNA by real time PCR with or without G-CSF-differentiation. Statistically significant differences (*p* < 0.01) in mRNA are indicated by *, **, ***, #, ## or ###. Non-significant differences are indicated by *p* value on the figure. Lysates from these cells were analyzed for protein expression by serially probing Western blots with antibodies to Calpastatin, Gas2 or Gapdh (loading control). Icsbp knockout (**B**) or Bcr-abl expression (**C**) increased Gas2 protein in myeloid progenitor cells and Calpastatin protein in differentiating granulocytes relative to control. (**D**) Gas2 expression was increased in human Lin^−^CD34^+^ CML cells, but Calpastatin was increased in differentiating CML granulocytes, in comparison to control. Lin^−^CD34^+^ bone marrow cells from CML or control subjects were analyzed for mRNA expression by real time PCR with or without G-CSF-differentiation. Statistically significant differences (*p* < 0.01) in mRNA were indicated by *, **, ***, #, ## or ###. Non-significant differences were indicated by ‘a’ or ‘b’.

We analyzed Gas2 and Calpastatin expression in these cells by quantitative real time PCR. G-CSF-differentiation significantly increased Calpastatin mRNA in control, Bcr-abl transduced and Icsbp^−/−^ cells (*p* < 0.01, *n* = 3; comparing CD34^+^ cells with versus without G-CSF in each group) (Figure 1A). This increase was significantly greater in Bcr-abl^+^ or Icsbp^−/−^ cells in comparison to control (*p* < 0.001, *n* = 3; comparing % increased expression in the three cell types). This was somewhat unexpected, since Icsbp was not known to influence Calpastatin expression. In contrast, Calpastatin expression was equivalent in myeloid progenitor cells from control, Bcr-abl^+^ and Icsbp^−/−^ mice (*p* = 0.1, *n* = 3).

We found significantly more Gas2 mRNA in Bcr-abl^+^ or Icsbp^−/−^ myeloid progenitor cells versus control cells, consistent with our prior studies (*p* < 0.001, *n* = 3; comparing the three cell types) (Figure 1A) [17]. However, this difference did not persist during granulocyte differentiation (*p* = 0.7, *n* = 3) (Figure 1A). In Western blots of lysate proteins, differences in Calpastatin or Gas2 protein expression between control versus Bcr-abl-transduced cells (Figure 1B) or control versus Icsbp^−/−^ cells (Figure 1C) were consistent with mRNA results.

We found a significant decrease in Icsbp mRNA expression in Bcr-abl^+^ myeloid progenitors and differentiating granulocytes, as anticipated (*p* < 0.001, *n* = 3; comparing control to Bcr-abl^+^ cells) (Figure 1A) [14–16]. In additional control studies, Icsbp was not expressed in Icsbp^−/−^ cells, and Bcr-abl was only detected in cells transduced with a Bcr-abl vector (Figure 1A).

We were interested in determining if these reciprocal, differentiation stage specific changes in Gas2 or Calpastatin expression were also found in the more complex genetic environment of human CML. To investigate this, we compared Lin^−^CD34^+^ bone marrow cells from CML subjects in chronic phase with Lin^-^CD34^+^ bone marrow cells from control subjects. Some cells were *ex vivo* differentiated with G-CSF. Similar to our studies of transduced murine bone marrow, Gas2 expression was significantly greater in Lin^-^CD34^+^ CML cells versus control Lin^−^CD34^+^ cells (*p* < 0.001, *n* = 3) and Calpastatin expression was significantly greater in CML cells undergoing granulocyte differentiation in comparison to similarly treated control cells (*p* < 0.001, *n* = 3) (Figure 1D).

These results suggested expression of Calpastatin and Gas2 was increased in Bcr-abl^+^ cells in a differentiation stage specific manner. We also found Icsbp contributed to regulation of Calpastatin expression in progenitor cells undergoing granulocyte differentiation. This implied Icsbp regulates Calpain activity throughout myelopoiesis, but through different mechanisms at various stages.

**Figure 2:**
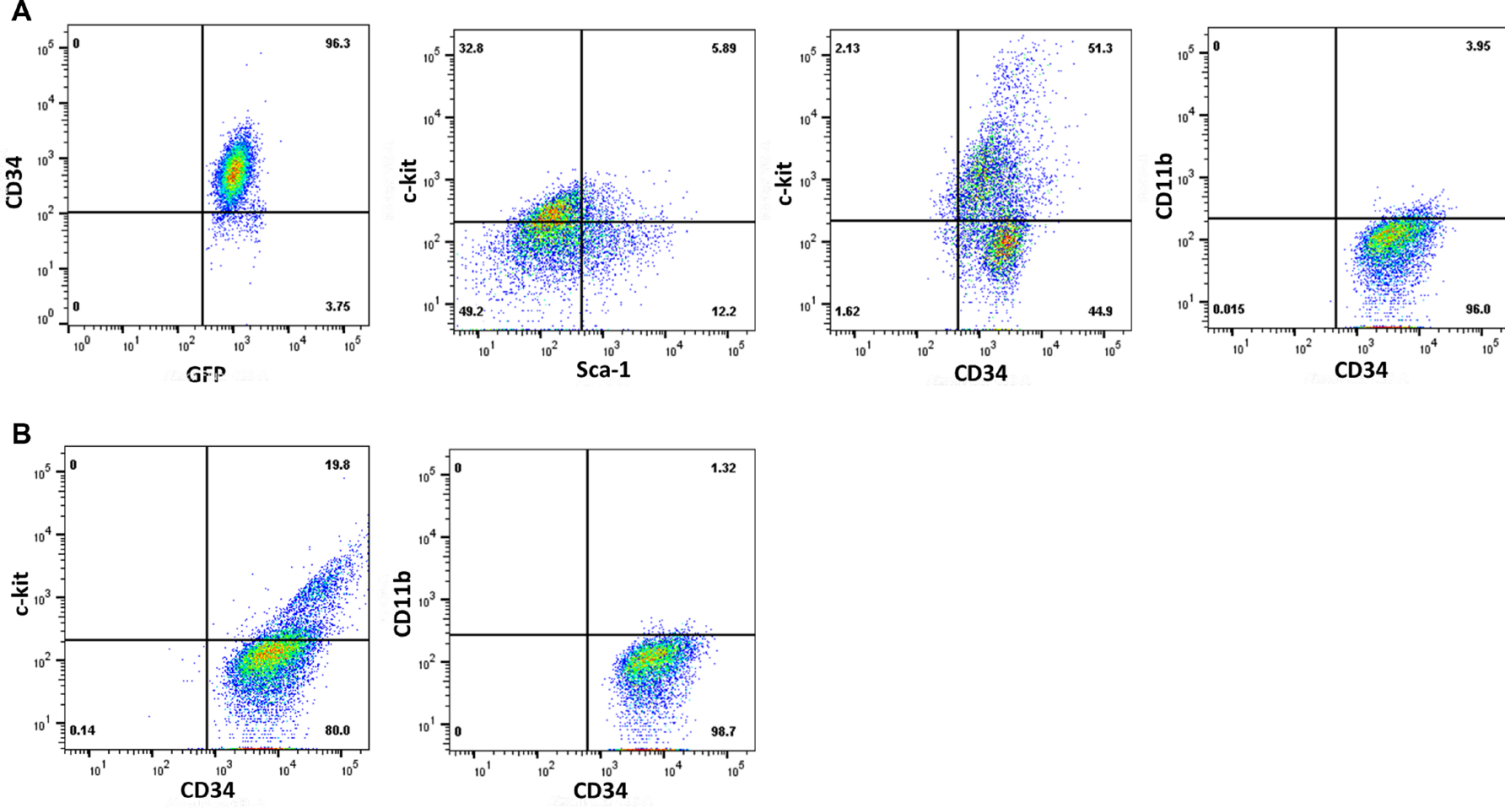
Characterization of CML cells by immuno-phenotyping Cells populations used in the experiments above were analyzed by immuno-phenotyping. Representative histograms are presented for (**A**). murine Bcr-abl transduced Lin^−^CD34^+^ bone marrow cells, or (**B**) human Lin^−^CD34^+^ bone marrow mononuclear cells from a CML subject in chronic phase.

To further characterize the cells used in these studies, we analyzed immuno-phenotype of Bcr-abl-transduced murine bone marrow myeloid progenitor cells and human CML cells. We found that Bcr-abl^+^ Lin^−^CD34^+^ murine bone marrow cells were also Sca1^-^ckit^+/−^CD11b^−^Gr1^−^ (Figure 2A). Similarly, the immuno-phenotype of human CML cells used in this study was ckit^+/−^CD34^+^CD11b^−^ (representative sample shown) (Figure 2B).

### Icsbp regulates CAST transcription in a differentiating myeloid progenitor cell

**Figure 3:**
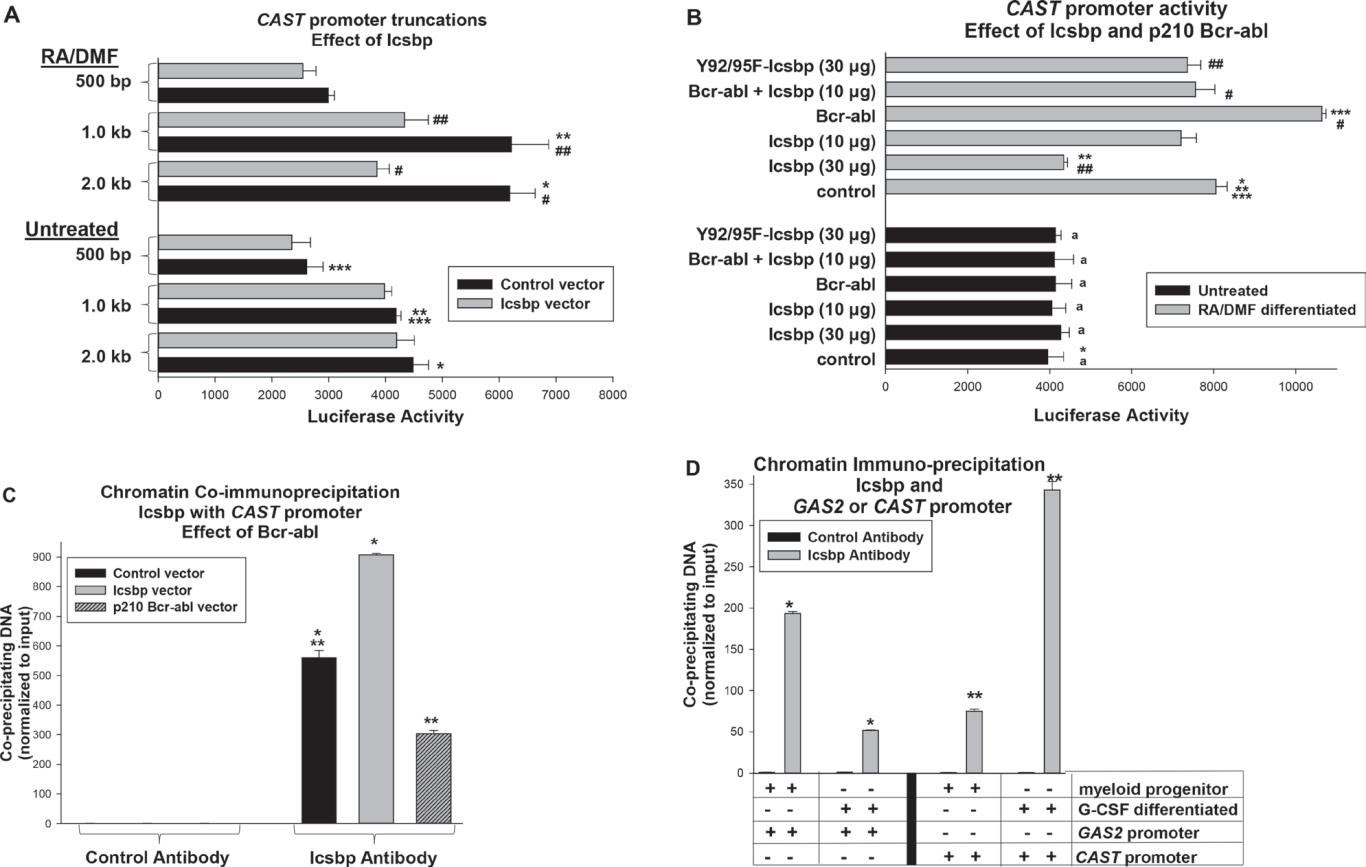
Icsbp represses the Calpastatin (*CAST*) promoter in differentiating myeloid cells (**A**) Icsbp repressed a cis element between −0.5 and −1.0 kb in the *CAST* 5′ flank. U937 cells were co-transfected with reporter constructs with various lengths of *CAST* 5′ flank and a vector to express Icsbp or control. Transfectants were assayed for reporter activity with or without granulocyte differentiation (with RA + DMF). Statistically significant differences (*p* < 0.01) were indicated by *, **, ***, #, or ##. (**B**) *CAST* promoter activity was repressed by Bcr-abl in an Icsbp-dependent manner and Icsbp repression required IRF-domain tyrosine residues. U937 cells were co-transfected with the −1.0 kb *CAST* promoter/reporter construct and vectors to express various combinations of Icsbp (wild type or Y-mutant) and Bcr-abl (or relevant control vectors). Reporter activity was determined as above. Statistically significant differences in reporter activity (*p* < 0.01) were indicated by *, **, ***, # or ##. Non-significant differences were indicated by ‘a’. (**C**) Bcr-abl decreased Icsbp binding to the *CAST* promoter. U937 cells were transfected with a vector to express Bcr-abl or Icsbp (or control vector) and analyzed by chromatin immunoprecipitation (ChIP) using antibody to Icsbp or irrelevant control antibody. Co-precipitating chromatin was amplified by real time PCR with primers flanking the *CAST* promoter sequence between −0.5 and −1.0 kb. Statistically significant differences (*p* < 0.01) were indicated by * or **. (**D**) Icsbp binding to the *CAST* promoter increased during granulocyte differentiation. CD34^+^ murine bone marrow cells were analyzed by ChIP with or without G-CSF-differentiation. Lysates were immuno-precipitated with an antibody to Icsbp or irrelevant control antibody, and co-precipitating chromatin was amplified by real time PCR using primers flanking Icsbp-binding cis elements in the *CAST* or *GAS2* genes. Statistically significant differences (*p* < 0.01) were indicated by * or**.

To clarify the role for Icsbp in Calpastatin expression in differentiating granulocytes, we investigated regulation of the *CAST* gene (encoding Calpastatin). We analyzed *CAST* promoter activity by transfecting U937 myeloid cells with a series of promoter/reporter constructs containing 2.0, 1.0 and 0.5 kb of *CAST* 5′ flank. Cells were co-transfected with an Icsbp expression vector (or control vector) and some cells were treated with retinoic acid (+ dimethyl formamide) to induce granulocyte differentiation [32]. We found Icsbp overexpression significantly decreased *CAST* promoter activity, but only in differentiating transfectants (*p* < 0.02, *n* = 6; differentiated transfectants with or without Icsbp vector) (Figure 3A). This effect required at least 1.0 kb of the *CAST* 5′ flank, identifying an Icsbp-influenced cis element between 500 bp and 1.0 kb (for the 0.5 kb *CAST* construct, *p* = 0.2, *n* = 6; reporter activity with or without Icsbp vector). Activity of either the 2.0 or 1.0 kb *CAST* promoter construct was significantly increased by differentiation (*p* < 0.01, *n* = 6 for either with versus without differentiation).

Since Icsbp becomes increasingly tyrosine phosphorylated during myelopoiesis, we examined the role of this post translational modification in differentiation stage specific *CAST* transcription. For these experiments, U937 cells were co-transfected with the 1.0 kb *CAST* promoter/reporter construct and a vector to express Icsbp with mutation of two tyrosine residues in the DNA-binding IRF domain to phenylalanine (Y92/95F-Icsbp). We previously found mutation of these residues impaired repression of other Icsbp target genes, including *GAS2* and *PTPN13* [12, 17]. Consistent with these prior results, Y92/95F-Icsbp did not effectively repress the *CAST* promoter in differentiating U937 transfectants (*p* = 0.4, *n* = 6; control versus Y92-95F-Icsbp) (Figure 3B). Wild type and Y-mutant Icsbp were equivalently expressed in U937 transfectants (data not shown) [33].

Since Bcr-abl decreases Icsbp expression in U937 transfectants, we investigated the effect of Bcr-abl on *CAST* promoter activity [13, 17]. We co-transfected U937 cells with the 1.0 kb *CAST* promoter/reporter construct and a vector to express Bcr-abl or control vector. We found Bcr-abl significantly increased *CAST* promoter activity in differentiated (*p* < 0.001, *n* = 3; with versus without Bcr-abl), but not undifferentiated, transfectants (*p* = 0.7, *n* = 3). To determine the influence of Icsbp on this Bcr-abl effect, we co-transfected cells with the *CAST* reporter construct and vectors to express Bcr-abl and Icsbp (or control vector). In preliminary studies, we identified an amount of Icsbp-overexpression that did not by itself influence *CAST* promoter activity in differentiated transfectants (*p* = 0.07, *n* = 3; control versus Icsbp vector) (Figure 3B). We found co-overexpression of Icsbp at this level significantly decreased Bcr-abl-induced *CAST* promoter activity (*p* < 0.01, *n* = 3; Bcr-abl versus Bcr-abl + Icsbp in differentiated cells) (Figure 3B).

We next investigated Icsbp interaction with the *CAST* promoter *in vivo* by chromatin immuno-precipitation using U937 transfectants with Bcr-abl, Icsbp or control vector. Cells were differentiated with retinoic acid and lysates analyzed for co-precipitation of the *CAST* promoter with Icsbp by real time PCR. We found Bcr-abl significantly decreased, and Icsbp-overexpression increased, co-precipitation of the *CAST* promoter with Icsbp (*p* < 0.001, *n* = 3; comparing either to control cells) (Figure 3C).

We also investigated interaction of endogenous Icsbp with the *CAST* or *GAS2* promoters in primary murine bone marrow cells. For these studies, bone marrow Lin^−^CD34^+^ cells were compared to cells undergoing *ex vivo* differentiation with G-CSF. Chromatin was co-precipitated from cell lysates with an Icsbp antibody (or irrelevant control) and amplified by real time PCR with primers flanking Icsbp binding cis elements in the two murine genes. We found Icsbp binding to the *CAST* promoter was significantly increased by G-CSF (*p* < 0.01, *n* = 3; myeloid progenitors versus differentiated cells) (Figure 3D). Conversely, Icsbp binding to the *GAS2* promoter was significantly decreased by G-CSF (*p* < 0.001, *n* = 3) (Figure 3D).

### The influence of Calpastatin or Gas2 on Calpain activity was differentiation stage specific

We previously identified Gas2 as the major Calpain inhibitor in myeloid progenitors, but not differentiating granulocytes [17]. The studies above suggested Calpastatin might influence Calpain activity in granulocytes, but not progenitor cells. To investigate this, we transduced wild type or Icsbp^−/−^ murine bone marrow cells with a lentiviral vector to express shRNAs specific to Calpastatin or Gas2 (or scrambled control vectors). Cells from wild type mice were also transduced with a Bcr-abl expression vector (or empty control vector). We analyzed Calpain activity in Lin^−^CD34^+^ transduced cells with or without G-CSF-induced differentiation.

**Figure 4:**
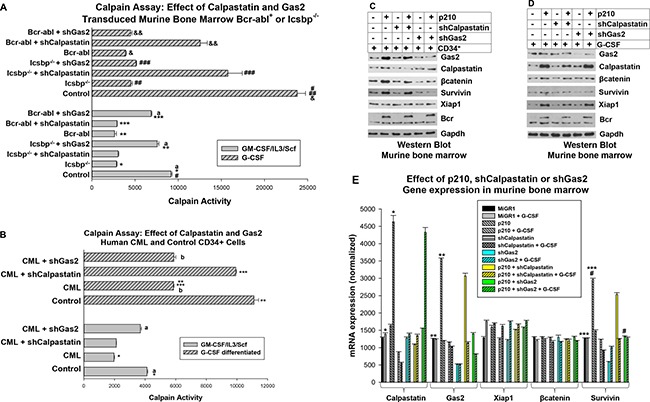
Calpastatin and Gas2 inhibit Calpain activity in CML in a differentiation stage specific manner (**A**) Calpastatin knockdown rescued Calpain activity in Bcr-abl^+^ or Icsbp^−/−^ myeloid progenitors undergoing granulocyte differentiation. Wild type or Icsbp^−/−^ murine bone marrow cells were transduced with vectors to express Calpastatin-specific shRNAs (or scrambled shRNA control). Some wild type cells were also transduced with a Bcr-abl expression vector. Lin^-^CD34^+^ cells were analyzed for Calpain activity with or without G-CSF-differentiation. Statistically significant differences (*p* < 0.02) were indicated by *, **, ***, #, ##, ###, & or &&, and non-significant differences by ‘a’. (**B**) Gas2 knockdown normalized Calpain activity in Lin^−^CD34^+^ CML cells and Calpastatin knockdown normalized Calpain activity in differentiating CML granulocytes. Human Lin^−^CD34^+^ bone marrow cells from CML or normal subjects were transduced with a retroviral vector to express Gas2 or Calpastatin specific shRNAs (or scrambled control). Cells were analyzed for Calpain activity with or without *ex vivo* G-CSF differentiation. Statistically significant differences (*p* < 0.01) were indicated by *, ** or ***, and non-significant differences by ‘a’ or ‘b’. (**C**) Knockdown of Gas2 increased βcatenin and Survivin protein in Bcr-abl-transduced myeloid progenitors. Murine bone marrow cells were transduced with vectors to express Bcr-abl (or control) and Gas2 or Calpastatin specific shRNAs. Lin^−^CD34^+^ cell lysates were analyzed by Western blots serially probed with antibodies to βcatenin, Survivin, Xiap1, Gas2, Calpastatin or Gapdh (loading control). (**D**) Some cells were similarly analyzed after G-CSF-differentiation. (**E**) Knockdown of Gas2 increased Survivin mRNA in Bcr-abl transduced murine myeloid progenitor cells. The cells described in ‘C’ and ‘D’ were also analyzed for mRNA expression by real time PCR. Statistically significant differences (*p* < 0.02) are indicated by *, **, *** or #.

We found significantly less Calpain activity in either Bcr-abl-transduced or Icsbp^−/−^ cells in comparison to control cells, with or without differentiation (*p* < 0.001, *n* = 6 for both comparisons). Knockdown of Calpastatin significantly increased Calpain activity in G-CSF differentiated Bcr-abl^+^ or Icsbp^−/−^ cells (*p* < 0.01, *n* = 6; with versus without Calpastatin shRNA), but did not increase Calpain activity in myeloid progenitor cells (*p* = 0.6, *n* = 6) (Figure 4A). In contrast, Gas2-knockdown significantly increased Calpain activity in Icsbp^−/−^ or Bcr-abl transduced myeloid progenitor cells (*p* < 0.001, *n* = 6; with versus without Gas2 shRNA), but did not alter Calpain activity in G-CSF-differentiated cells (*p* > 0.1, *n* = 6) (Figure 4A). Calpain activity in Icsbp^−/−^ or Bcr-abl^+^ myeloid progenitor cells expressing Gas2-shRNA was not significantly different than in control cells (*p* = 0.2, *n* = 6; comparing the three groups).

We also investigated the impact of Gas2 or Calpastatin on Calpain activity in human Lin^−^CD34^+^ bone marrow cells from CML or control subjects. For these studies, Lin^−^CD34^+^ cells were transduced with lentiviral vectors to express shRNAs specific to Gas2 or Calpastatin (or scrambled control vectors). Cells were assayed for Calpain activity with or without *ex vivo* G-CSF differentiation. We found significantly less Calpain activity in CML cells versus control, with or without G-CSF (*p* < 0.01, *n* = 3; control versus CML). Calpain activity in Lin^-^CD34^+^ CML cells with Calpastatin knockdown was not significantly different than control Lin^−^CD34^+^ cells (*p* = 0.6, *n* = 3), but Calpastatin knockdown rescued Calpain activity G-CSF differentiated CML cells (Figure 4B). Conversely, knockdown of Gas2 did not influence Calpain activity in G-CSF differentiated CML cells, but significantly increased Calpain activity in Lin^−^CD34^+^ CML cells (*p* < 0.01, *n* = 3; control versus CML) (Figure 4B).

### Gas2 and Calpastatin influence βcatenin/Survivin and Xiap1 in a differentiation stage specific manner

Results above indicate Bcr-abl impairs Calpain activity in Lin^−^CD34^+^ myeloid progenitors (functional LSCs) through increased Gas2 expression, and in G-CSF-differentiated cells (differentiating progenitors and CML granulocytes) through increased Calpastatin. This decrease in Calpain activity might influence a common set of substrates in all myeloid cells, or there might be differential influence on Calpain substrates during granulopoiesis. Since we were interested in apoptosis resistance in CML-LSCs, we investigated the impact of decreased Calpain activity in Bcr-abl^+^ cells on two relevant IAPs. Xiap1 is a direct Calpain substrate and Survivin is the target gene of βcatenin; another Calpain substrate [19, 34].

For these experiments, we co-transduced murine bone marrow cells with vectors to express Bcr-abl and shRNAs specific to Gas2 or Calpastatin (or scrambled control vectors). Lysate proteins from Lin^−^CD34^+^ cells or G-CSF-differentiated cells were analyzed by Western blot. We found increased expression of βcatenin and Survivin in Bcr-abl transduced Lin^−^CD34^+^ cells versus control (Figure 4C), but this was much less pronounced after G-CSF differentiation (Figure 4D). Conversely, expression of Xiap1 was increased in G-CSF differentiated, Bcr-abl transduced cells in comparison to control cells, but not in myeloid progenitor cells (Figure 4C and 4D). Knockdown of Gas2 decreased βcatenin/Survivin in Bcr-abl^+^ Lin^−^CD34^+^ bone marrow cells (Figure 4C), but not in cells differentiated with G-CSF (Figure 4D). Knockdown of Calpastatin had the opposite effect; Xiap1 protein was decreased in G-CSF differentiated cells, but not altered in myeloid progenitors.

To clarify mechanisms for this, we determined the effect of Gas2 or Calpastatin knockdown on mRNA expression of βcatenin, Survivin and Xiap1. We found Bcr-abl expression significantly increased Calpastatin mRNA in G-CSF differentiated cells and Gas2 mRNA in Lin^−^CD34^+^ cells (*p* < 0.001, *n* = 3; for either comparison, Bcr-abl versus control) (Figure 4E). Bcr-abl expression also significantly increased Survivin mRNA in Lin^−^CD34^+^ cells (*p* < 0.001, *n* = 3), but not G-CSF treated cells. This was consistent with an effect of Bcr-abl on βcatenin/Survivin in myeloid progenitors, but not differentiating granulocytes. Knockdown of Gas2 (but not Calpastatin) reversed this Bcr-abl induced increase in Survivin expression, consistent with the effect on βcatenin protein (in Figure 4C). Although βcatenin protein was decreased by Gas2 knockdown in Lin^−^CD34^+^ cells (Figure 4C), and Xiap1 protein by Calpastatin knockdown in differentiating cells (Figure 4D), expression of Xiap1 or βcatenin mRNA was not significantly altered by Bcr-abl or Calpastatin or Gas2 knockdown (Figure 4E). These results suggested βcatenin and Xiap1 are stabilized differentiation stage specific Calpain substrates.

### Survivin and Xiap1 have differentiation stage specific effects on apoptosis in Bcr-abl expressing cells

The results in the previous section suggested apoptosis resistance of CML-LSCs might be due to a βcatenin-dependent increase in Survivin, but increased Xiap1 might contribute to apoptosis resistance in circulating CML cells or differentiating progenitors. This would be an important distinction, because it implies targeting Survivin would be more effective than targeting Xiap1 to abolish LSCs during TKI treatment. To investigate this, we first transduced Icsbp^−/−^, Bcr-abl-transduced or control murine bone marrow cells with a vector to express Calpastatin-specific shRNAs (or scrambled control shRNAs). Lin^−^CD34^+^ myeloid progenitor cells were compared to cells differentiated with G-CSF. Cells were assayed for apoptosis by flow cytometry for Annexin V staining, with or without pre-treatment with a Fas-agonist antibody [12, 13].

**Figure 5:**
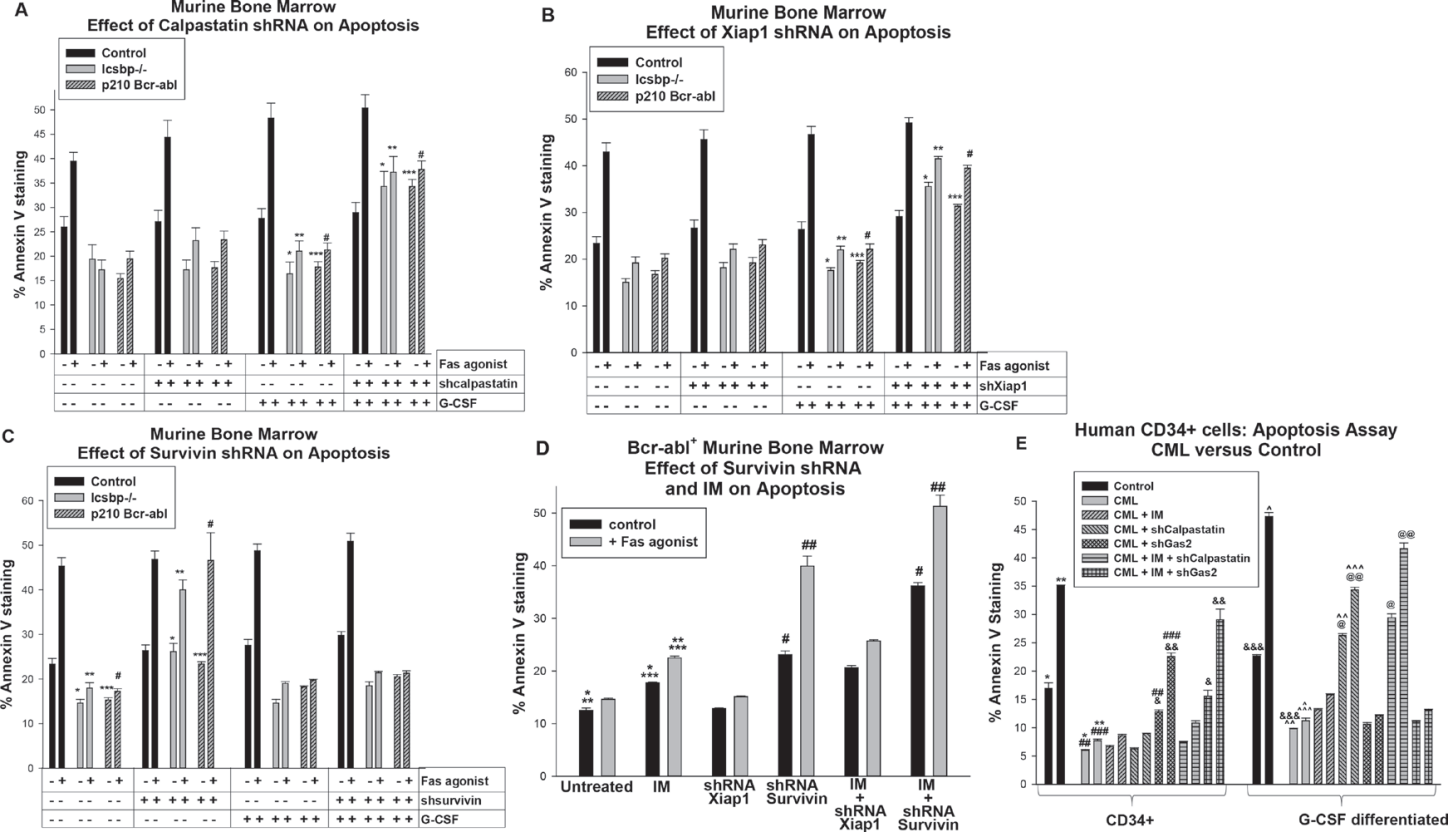
Apoptosis resistance in Bcr-abl^+^ or Icsbp deficient myeloid progenitor cells was reversed by knockdown of Survivin; apoptosis resistance in Bcr-abl^+^ or Icsbp deficient granulocytes was reversed by knockdown of Calpastatin or Xiap1 Wild type or Icsbp^−/−^ murine bone marrow cells were transduced with a vector to express a shRNA specific to Calpastatin, Xiap1 or Survivin (or scrambled shRNA control). Some wild type cells were co-transduced with a Bcr-abl-expression vector (or control). Lin^−^CD34^+^ cells were analyzed for apoptosis by Annexin V staining, with or without G-CSF-differentiation, with or without Fas-agonist antibody. (**A**) Knockdown of Calpastatin increased sensitivity to intrinsic and Fas-induced apoptosis in differentiating Bcr-abl transduced or Icsbp^−/−^ myeloid progenitor cells. Statistically significant differences (*p* < 0.02) were indicated by *, **, *** or #. (**B**) Knockdown of Xiap1 increased sensitivity to intrinsic and Fas-induced apoptosis in differentiating Bcr-abl transduced or Icsbp^−/−^ myeloid progenitors. Statistically significant differences (*p* < 0.02) were indicated by *, **, *** or #. (**C**) Knockdown of Survivin increased sensitivity to intrinsic and Fas-induced apoptosis in Bcr-abl transduced or Icsbp^-/-^ myeloid progenitors. Statistically significant differences (*p* < 0.02) were indicated by *, **, *** or #. (**D**) Imatinib (IM) enhanced the effect of Survivin knockdown on apoptosis in Bcr-abl-transduced murine myeloid progenitor cells. Some Lin^−^CD34^+^ cells transduced with vectors to express Bcr-abl and shRNA to Xiap1 or Survivin (or scrambled control vector) were treated with IM prior to analysis. Statistically significant differences (*p* < 0.02) were indicated by *, **, ***, # or ##. (**E**) IM enhanced effects of Gas2 knockdown on apoptosis in human Lin^−^CD34^+^ CML bone marrow cells. Human Lin^−^CD34^+^ bone marrow cells from CML or control subjects were transduced with vectors to express shRNA specific to calpastatin or Gas2 (or scrambled shRNA control) and analyzed for apoptosis, with or without treatment with IM, and with or without Fas-agonist. Statistically significant differences (*p* < 0.02) were indicated by *, **, ***, #, ##, ###, &, &&, &&&, ^, ^^, ^^^, @ or @@.

In comparison to control cells, either Bcr-abl-expression or Icsbp-knockout significantly decreased both intrinsic (*p* < 0.01, *n* = 6; comparing the three groups) and Fas-induced (*p* < 0.001, *n* = 6) apoptosis in myeloid progenitor populations, consistent with our previous studies (Figure 5A) [12, 13]. These differences persisted after G-CSF-differentiation (*p* < 0.01, *n* = 6; comparing the three groups). In Bcr-abl transduced or Icsbp^-/-^ progenitor cells, knockdown of Calpastatin did not significantly alter intrinsic apoptosis (*p* > 0.3, *n* = 6; with versus without Calpastatin shRNA) or Fas-induced apoptosis (*p* > 0.4, *n* = 6; with versus without Calpastatin shRNA) (Figure 5A). However, knockdown of Calpastatin in G-CSF-differentiated Bcr-abl^+^ or Icsbp^-/-^ cells significantly increased both intrinsic and Fas-induced apoptosis (*p* < 0.001, *n* = 6; with versus without Calpastatin shRNA) (Figure 5A). Intrinsic apoptosis was normalized by Calpastatin knockdown these cells (*p* = 0.2, *n* = 6; comparing the three groups). However, calpastatin knock-down did not completely normalize Fas-induced apoptosis in G-CSF differentiated, Bcr-abl^+^ or Icsbp^-/-^ cells (*p* = 0.02, *n* = 6). And, Calpastatin knockdown did not alter apoptosis in control cells with or without G-CSF (*p* > 0.5, *n* = 6).

These results identified functional impact of Calpastatin in Bcr-abl expressing granulocytes. To determine if increased Xiap1 contributed to this effect, control, Bcr-abl-transduced and Icsbp^−/−^ murine bone marrow cells were transduced with Xiap1 specific shRNA vectors (or scrambled control). Lin^−^CD34^+^ cells were analyzed for apoptosis, with or without G-CSF-differentiation, and with or without a Fas-agonist. We found a significant increase in both intrinsic and Fas-induced apoptosis in Xiap1-shRNA transduced, G-CSF-differentiated Bcr-abl^+^ or Icsbp^−/−^ cells *p* < 0.01, *n* = 6; with versus without Xiap1 knockdown) (Figure 5B). This increase was not observed in experiments with myeloid progenitor cells. As for Calpastatin knockdown, the relative increase in intrinsic apoptosis with Xiap1 knockdown was greater than the increase in Fas-induced apoptosis. These results were consistent with the influence of Calpastatin on Calpain activity, and therefore Xiap1, specifically in differentiating myeloid progenitors and/or granulocytes.

We performed similar studies to determine if Survivin exhibited differentiation stage specific effects on apoptosis in Bcr-abl expressing or Icsbp-deficient murine bone marrow cells. For these studies, wild type, Bcr-abl-transduced and Icsbp^−/−^ murine bone marrow cells were transduced with a vector to express Survivin specific shRNAs (or scrambled control shRNAs) and analyzed for apoptosis as above. We found knockdown of Survivin did not influence intrinsic or Fas-induced apoptosis in G-CSF differentiated cells (*p* > 0.1, *n* = 6; with versus without Survivin shRNA) (Figure 5C). However, knockdown of Survivin increased intrinsic apoptosis (*p* < 0.01, *n* = 6; with versus without Survivin shRNAs) and Fas-induced apoptosis (*p* < 0.001, *n* = 6) in Bcr-abl^+^ or Icsbp^−/−^ myeloid progenitor cells (Figure 5C). In these cells, Survivin knockdown normalized intrinsic and Fas-induced apoptosis (*p* > 0.4, *n* = 6; comparing the three groups for either intrinsic or Fas-induced apoptosis). Knockdown of Survivin did not alter apoptosis in control cells, with or without G-CSF-differentiation (*p* > 0.3, *n* = 6). Knockdown of Survivin mimicked our prior results with Gas2-knockdown in Bcr-abl-transduced or Icsbp-knockout myeloid progenitor cells [17].

Based on these results, we investigated the impact of IM on apoptosis in Bcr-abl^+^ Lin^−^CD34^+^ murine bone marrow cells. For these studies, myeloid progenitor cells were analyzed with or without expression of shRNAs specific to Xiap1 or Survivin, with or without treatment with IM. We found IM treatment significantly augmented apoptosis, with or without Fas agonist treatment, in Bcr-abl-transduced cells (*p* < 0.02, *n* = 3) (Figure 5D). IM treatment also significantly augmented Fas-induced apoptosis in Bcr-abl^+^ myeloid progenitors expressing Survivin specific shRNAs (*p* < 0.01, *n* = 3) (Figure 5D). Indeed, Fas-induced apoptosis in IM treated Bcr-abl^+^ myeloid progenitor cells with Survivin knockdown was slightly greater than in control myeloid progenitor cells. In contrast, the combination of Xiap1 specific shRNA and IM was not significantly different than IM alone (*p* = 0.08, *n* = 3) (Figure 5D).

We performed similar studies to determine the impact of IM on calpain-related apoptosis in human CML bone marrow myeloid progenitor cells. For these experiments, Lin^−^CD34^+^ bone marrow cells from human CML or control subjects were studied with or without G-CSF differentiation, and with or without Fas-agonist. Some CML cells were transduced with vectors to express shRNAs specific to Gas2 or Calpastatin. We found IM treatment significantly increased apoptosis in CML-myeloid progenitors with Gas2 knockdown, with or without Fas-agonist (*p* < 0.001, *n* = 3), and in differentiating CML granulocytes with Calpastatin knockdown, with or without Fas-agonist (*p* < 0.01, *n* = 3) (Figure 5E).

## DISCUSSION

Persistence of LSCs during treatment prevents cure of CML by TKIs in the majority of patients, and permits accumulation of additional mutations leading to overt drug resistance or BC [4, 6–8]. Our previous studies suggested resistance to Fas-induced apoptosis might contribute to this effect [12–14]. In other previous studies, we found decreased Calpain activity in Bcr-abl^+^ myeloid progenitor cells due to decreased Icsbp expression and consequent increased expression of Gas2, a Calpain inhibitor [17]. Decreased Calpain activity in these cells stabilized βcatenin protein and increased expression of βcatenin target genes, including Survivin, an Inhibitor of Apoptosis Protein (IAP) [17]. Although Gas2 expression decreased during the course of normal myelopoiesis, we found Icsbp did not repress *GAS2* transcription in granulocytes [17]. Therefore, Calpain activity was decreased in both Bcr-abl^+^ myeloid progenitors and differentiating granulocytes, but through different mechanisms. Our first goal in the current study was to define mechanisms for Calpain regulation throughout myelopoiesis and the relative contribution of these mechanisms to apoptosis resistance in CML-LSCs.

Calpain has substrates in addition to βcatenin that may contribute to the pathogenesis of CML, including Xiap1, Stat3 and Stat5 [19, 35]. Increased expression of Xiap1 would be anticipated to influence apoptosis resistance in CML, but this has not been previously investigated. It is also unknown if Calpain influences the same substrates throughout myelopoiesis, or if there are differentiation stage specific substrate preferences. In the current work, we investigated the relative roles of the Calpain substrates βcatenin/Survivin and Xiap1 in apoptosis resistance in CML.

**Figure 6:**
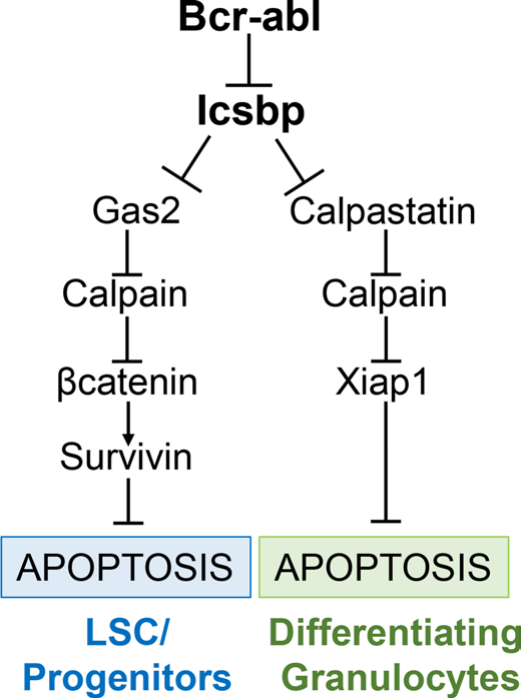
Graphical representation of regulation of apoptosis in Bcr-abl^+^ cells by Icsbp, Gas2 or Calpastatin, Calpain, and Xiap1 or βcatenin/Survivin

We found Bcr-abl influenced Calpain activity in differentiating granulocytes through decreased expression of Calpastatin, another Calpain inhibitor. Icsbp repressed the *CAST* promoter under these conditions, identifying it as another Calpain-relevant Icsbp target gene. This was consistent with our prior studies indicating Calpastatin (not Gas2) was the major Calpain inhibitor in mature phagocytes [17]. Therefore, Icsbp regulated Calpain activity by differentiation stage specific regulation of two separate target genes (Figure 5). Consistent with this, we determined Calpain activity was decreased in CD34^+^ cells and differentiating granulocytes from CML subjects in comparison to control cells. The influences of Gas2 and Calpastatin in human CML paralleled those in Bcr-abl-transduced murine bone marrow. We also found differentiation stage specific regulation of apoptosis by Survivin versus Xiap1 in Bcr-abl^+^ cells (Figure 6). Specifically, Survivin knockdown increased intrinsic and Fas-induced apoptosis in Bcr-abl^+^ CD34^+^ cells, but not differentiating CML-granulocytes. Conversely, Xiap1 knockdown increased apoptosis in Bcr-abl^+^ cells undergoing G-CSF differentiation. This was associated with greater expression of Survivin in myeloid progenitors and of Xiap1 in differentiating granulocytes.

Icsbp was hypothesized to be a CML leukemia suppressor based on expression profiling of human leukemia subjects [24, 25]. This hypothesis was validated in various murine models, including mice transplanted with Bcr-abl-transduced bone marrow cells, with or without re-expression of Icsbp, and mice with *IRF8* gene disruption [26–28]. To understand the role of Icsbp in leukemogenesis, it is useful to define normal functions for this transcription factor during myelopoiesis.

In our prior studies, we identified three discrete functions for Icsbp with implications for leukemogenesis. First, Icsbp activates transcription of genes encoding phagocyte effector proteins during the innate immune response [33]. Second, Icsbp reverses expansion of hematopoietic stem cells (HSC) and myeloid progenitor cells occurring during the emergency granulopoiesis response, and is required to re-set granulopoiesis to steady state levels [36]. Our studies suggested the latter requires repression of genes encoding Gas2 and Fap1 by Icsbp [36]. Third, Icsbp increases expression of core components of the Fanconi DNA repair pathway during emergency granulopoiesis (*FANCC* and *FANCF*) [37, 38]. Rapid expansion of hematopoietic stem and myeloid progenitor cells during emergency granulopoiesis involves S phase shortening and relative Fas-resistance. Therefore, genotoxic stress and a risk of accumulating cells with DNA damage are characteristics of emergency granulopoiesis. Decreased Icsbp expression would contribute to several events involved in the pathogenesis of CML, including myeloid progenitor expansion, susceptibility to DNA-damage, and phagocyte functional incompetence.

The results of our current studies imply Gas2/βcatenin/Survivin would be more effective therapeutic targets for enhancing apoptosis in CML-LSCs, but Calpastatin/Xiap1 inhibition would promote apoptosis in circulating CML cells (Figure 5). This is of more than theoretical importance, since small molecule inhibitors of Survivin or Xiap1 are already in human clinical trials for solid tumors [21, 23]. Additional studies identifying the functional impact of these inhibitors on TKI resistance and LSC persistence in pre-clinical models are planned.

## MATERIALS AND METHODS

### Plasmids

p210-Bcr-abl in MiGR1 was obtained from Dr. Ravi Bhatia (University of Alabama, Birmingham). The Icsbp cDNA was obtained from Dr. Ben Zion-Levi (Technion, Haifa, Israel) and the full length cDNA generated by PCR and subcloned into the mammalian expression vector pcDNA (Stratagene, La Jolla, CA), as described [33]. A tyrosine mutant form of the Icsbp cDNA with mutation of two tyrosine residues in the IRF DNA-binding domain (Y92F-Y94F) was generated as previously described [33]. Plasmids with three different shRNAs specific to murine or human Gas2, Calpastatin, Survivin or Xiap1 (and control scrambled sequences) in the pRS retroviral vector were obtained from Origene (Origene USA, Rockville MD).

To generate reporter constructs, several sequences from the 5′ flank of the *CAST* gene were obtained by PCR from U937 genomic DNA. Fragments were sequenced on both strands and compared to established databases of genomic sequences. Constructs were generated in the pGL3-basic reporter vector (Promega) using 2.0 kb, 1.0 kb, or 500 bp of *CAST* 5′ flank.

### Myeloid cell line culture

The human leukemia cell line U937 [39] was obtained from Andrew Kraft (University of Arizona, Tucson). Cells were maintained as described [33]. For granulocyte differentiation, cells were treated for 48 hrs with retinoic acid with the addition of dimethyl formamide for the last 24 hours (Sigma-Aldrich Inc., St. Louis, MO) [32].

### Murine bone marrow cells

All murine studies were performed with approval of the Animal Care and Use Committees of Northwestern University and Jesse Brown VA Medical Center. Mice with disruption of the *IRF8* gene (Icsbp^−/−^ mice) were obtained from Dr. Keiko Ozato (NIH, Bethesda, MD) [27].

Mononuclear cells were isolated from femurs of wild type or Icsbp^−/−^ C57/BL6 mice and cultured in DME supplemented with 10% FCS, 1% pen-strep, 10 ng/ml GM-CSF, 10 ng/ml IL-3, 100 ng/ml Scf (R&D Systems Inc., Minneapolis MN). Lin^-^ckit^+^CD34^+^ cells were isolated from these cultures by magnetic bead affinity technique (Miltenyi Biotechnology, Auburn, CA) (referred to as “myeloid progenitor conditions”). Lin^-^ckit^+^CD34^+^ cells (with increased βcatenin expression) represent the LSC population in murine CP-CML [27]. Some cells were differentiated with 10 ng/ml G-CSF for 24 hrs (R&D Systems Inc., Minneapolis MN) (> 70% of cells are Gr1^+^ under these conditions; not shown).

Bcr-abl retrovirus and shRNA expressing lentiviruses were prepared by transfecting 293T cells with the relevant plasmid and the Ecopack plasmid [14]. Viral supernatants collected 48 hours post-transfection were titered in NIH3T3 cells. Murine bone marrow cells were transduced by incubation with viral supernatant (~10^7^ pfu/ml) supplemented with polybrene (6 μg/ml) [14]. Transgene expression was confirmed by PCR and/or %GFP^+^ cells.

### Human bone marrow transduction

All human studies were performed under the auspices of approved protocols by the IRB of Northwestern University and Jesse Brown VA in accordance with an assurance filed with and approved by the U.S. Department of Health and Human Services. Bone marrow for these studies was obtained at the time of a clinically indicated bone marrow aspiration. CD34^+^ cells were separated from total bone marrow mononuclear cells using the Miltenyi magnetic bead antibody affinity technique, as described for murine bone marrow cells. Lin^−^CD34^+^ normal human bone marrow cells purchased from Stem Cell Technologies (Vancouver, Canada) to use as controls for these studies. All human Lin^−^CD34^+^ cells were cryopreserved under the same conditions and recovered for analysis. Cells were transduced with viral vectors as described for primary murine bone marrow cells.

### Quantitative real time PCR

RNA was isolated with Triazol reagent and tested for integrity by electrophoresis. Primers were designed with Applied Biosystems software (Grand Island NY) and PCR performed by SYBR green method. Result were normalized to 18S and actin. At least three independent samples were tested in triplicate for all experiments (reported as *n* = 3).

### Western blots

Cells were lysed in SDS sample buffer, separated by SDS-PAGE, transferred to nitrocellulose and serially probed with antibodies to Gas2 (Proteintech, Rosemont, IL), Survivin (Proteintech), Xiap1 (Proteintech), Calpastatin (Santa Cruz Biotechnology, Santa Cruz, CA), Icsbp (Santa Cruz Biotech), βcatenin (BD Biosciences, San Jose, CA) or Gapdh (as a loading control). Experiments were repeated at least twice with different lysates and representative blots are shown.

### Transient transfections for CAST promoter activity

U937 cells (32 × 10^6^/ml) were transfected with various combinations of vectors to express Icsbp (wild type or Y92F-Y95F mutant), Bcr-abl or control vector. Cells were co-transfected with a firefly-luciferase reporter construct with various sequences from the proximal *CAST* 5′ flank and a CMV/renilla reporter (to control for transfection efficiency). Constructs with *CAST* promoter sequences were compared to empty, control reporter vector in these experiments. Dual luciferase assays were performed as per manufacturer's instructions (Promega) as previously described [12, 13, 17]. Some transfectants were treated with retinoic acid + dimethyl formamide to induce granulocyte differentiation.

### Flow cytometry

Bone marrow or peripheral blood was analyzed for GFP-expression on a Becton-Dickinson FACScan (Cambridge, MA). For determination of apoptosis, cells were incubated for 24 hours with Fas-agonist antibody (5 μg/ml CH11; BD Bioscience Inc., San Jose CA), labeled with PE-conjugated CD34 antibody and analyzed by Annexin V-Apoptosis Detection Kit I (eBioscience, San Diego CA).

### Chromatin co-immunoprecipitation

Cells were incubated briefly in media supplemented with formaldehyde and lysates were sonicated to generate chromatin fragments with an average size of 0.5 kb [40]. Lysates were immuno-precipitated with Icsbp antibody or irrelevant control anti-GST antibody (Santa Cruz Biotechnology). Specific co-precipitation of *GAS2* or *CAST* promoter sequences was determined by quantitative PCR analysis using the standard curve method. For these experiments, primers were designed to flank the Icsbp binding cis elements in these promoters. Control studies were performed with primers to one intron sequence and one region of the coding sequence.

### Calpain activity assays

Calpain activity was determined using a commercially available “Calpain-Glo” assay kit (Promega, Madison, WI). Cell lysates were assayed using the fluorescent labeled substrate provided in the kit and calpain activity determined by flurometric change.

## Abbreviations

AML: acute myeloid leukemia
CML: chronic myeloid leukemia
Fap1: Fas associated phosphatase 1
Gas2: Growth Specific Arrest 2
G-CSF: granulocyte colony stimulating factor
GM-CSF: granulocyte monocyte colony stimulating factor
GMP: granulocyte monocyte progenitor
HSC: hematopoietic stem cell
IAP: inhibitor of apoptosis protein
Icsbp: interferon consensus sequence binding protein
shRNA: short hairpin RNA
Xiap1: X-linked inhibitor of apoptosis protein 1.
